# Quantitative analysis of vascular calcification

**DOI:** 10.3892/etm.2013.1385

**Published:** 2013-11-06

**Authors:** JIN HYUN JOH, DONG IK KIM

**Affiliations:** 1 Department of Surgery, Kyung Hee University Hospital at Gangdong, Kyung Hee University School of Medicine, Seoul 134-727, Republic of Korea; 2 Division of Vascular Surgery, Department of Surgery, Samsung Medical Center, Sungkyunkwan University School of Medicine, Seoul 135-710, Republic of Korea

**Keywords:** vascular calcification, mineral components, calcium, phosphate

## Abstract

Vascular calcification is a prominent feature of atherosclerosis. The mineral composition and quantity within calcified arterial plaques remains unelucidated; therefore, the aim of this study was to analyze the mineral composition of such plaques. Calcified arterial plaques were obtained from patients with abdominal aortic aneurysms (AAAs) and carotid artery stenoses. Calcified aneurysmal plaques were obtained during the routine open repair of AAAs, while calcified carotid plaques were collected from patients who underwent carotid endarterectomy (CEA). Following the appropriate preparation of each sample, inductively coupled plasma atomic emission spectrometry (ICP-AES) was used to analyze the calcium and phosphate levels, while flame atomic absorption spectrometry (FAAS) was used to analyze the levels of iron and zinc. The levels of these mineral components were evaluated. In the aortic and carotid plaques, the mean calcium concentration was 9.83 and 11.94 wt.%, respectively, and the mean phosphate concentration was 4.31 and 6.08 wt.%, respectively. It was not possible to analyze the absolute concentration of iron in the carotid plaques due to the concentration being below the measurement limit. The zinc concentration was variable between samples. In conclusion, the main components of aortic and carotid plaques are calcium and phosphate. The mineral concentrations of the plaques in the present study may be used as reference values for further studies on vascular calcification. More studies are required to elucidate the correlation between the mineral components and vascular calcification.

## Introduction

Vascular calcification, the deposition of calcium phosphate in the vessel wall, is a prominent feature of atherosclerosis. Calcification may occur in the intima and media of the vessel wall with aging, diabetes or uremia (1).

Although the mechanism underlying vascular calcification has been defined through *in vitro* studies and molecular biological techniques, unanswered questions remain, particularly with respect to the correlation between bone formation and vascular calcification. Bone formation is an active process involving the production of an extracellular matrix conducive to mineralization. Arterial calcification was previously considered to be a degenerative, end-stage process of vascular diseases, and early studies of arterial calcification described an association with tissue necrosis (2). An apoptotic mechanism of cell death has also been shown to contribute to arterial calcification (3–5). The presence of proteins involved in bone formation, such as morphogenetic proteins, osteonectin, osteocalcin and matrix GLA protein, in calcified vascular tissues suggests that vascular calcification is an organized, regulated process, similar to mineralization in bone tissue (6).

Calcium and phosphate are the main mineral components of human bone. However, the mineral composition and quantity within calcified arterial plaques remain unelucidated. The aim of this study was to evaluate the mineral composition in calcified arterial plaques.

## Materials and methods

This study was approved by the Samsung Medical Center, Sungkyunkwan University School of Medicine Institutional Review Board (Seoul, Korea). Calcified arterial plaques were obtained from patients with abdominal aortic aneurysms (AAAs) and carotid artery stenoses. Calcified aneurysmal plaques were obtained during the routine open repair of AAAs using conventional techniques. The conventional repair of the AAAs included closing the AAA sac over the graft to protect the prosthetic graft from eroding into the bowel and from exposure to intraperitoneal contamination. Prior to the closure of the AAA sac, a piece of calcified aneurysmal plaque was obtained. Calcified carotid plaques were collected from patients who underwent carotid endarterectomy (CEA). CEA was performed using conventional surgical techniques. Briefly, the carotid artery was incised and the plaques were removed from the lumen as a single specimen. A piece of specimen was obtained from the most calcified portion of the endarterectomized plaques. Patients with carotid artery stenoses or AAAs whose lesions demonstrated heavy calcification when analyzed by preoperative computed tomography (CT) scanning were selected for the study. The informed consent was obtained from either the patients or the patients’ family.

All plaques were immediately rinsed with normal saline to remove the blood components, prior to digestion being conducted for each sample. This process was performed in a closed vessel with high-pressure at 1,200 psi, heated at 200°C by microwave energy. The 5 ml of 0.1 M nitric acid was used to digest 1 g of each sample.

Certain mineral components of the plaques, specifically, calcium, phosphate, iron and zinc, were investigated. Inductively coupled plasma atomic emission spectrometry (ICP-AES; Varian-720-ES, Agilent, Santa Clara, CA, USA) was used to analyze the calcium and phosphate levels. The inductively coupled plasma was an electrically conductive gaseous mixture of argon, argon ions and electrons. Plasma was produced from a stream of argon gas, which was then energized by a high-energy, radio-frequency field. The sample was subsequently introduced as an aerosol mixed with argon into the plasma, where high temperature caused efficient desolvation, volatilization, atomization, excitation and ionization of the samples (7,8).

Flame atomic absorption spectrometry (FAAS; ICE 3000 Series, ThermoScientific, Rockford, IL, USA) was used to analyze the iron and zinc levels. In the FAAS, a flame was used to atomize a compound in solution. The air-acetylene flame was used in atomic absorption spectrometry. FAAS has been indicated to be the simplest and easiest analytical technique for element analysis (9,10).

## Results

A total of 11 pieces of aortic plaque were obtained from 11 patients during the routine open repair of AAAs. Six (n=6) pieces of carotid plaque were obtained from six patients during CEA.

The levels of the mineral components calcium, phosphate, iron and zinc, were analyzed in each sample. Table I summarizes the concentration of calcium in each of the plaques. The calcium concentration ranged from 2.10 to 19.3 wt.% in aortic plaques and from 4.43 to 18.2 wt.% in carotid plaques. The mean calcium concentration of the aortic and carotid plaques was 9.83 and 11.94 wt.%, respectively. The mean calcium concentration of the aortic plaques was lower than that of the carotid plaques. Table II shows the phosphate concentration in each sample. The mean phosphate concentration in the aortic plaques was 4.31 wt.% (range, 0.97–8.53 wt.%). In the carotid plaques, the mean value was 6.08 wt.% (range, 4.28–8.33 wt.%). Similar to the calcium concentration, the mean phosphate concentration of the aortic plaques was lower than that of the carotid plaques. Tables III and IV, respectively, show the concentrations of iron and zinc. The mean concentration of iron was 0.0067 wt.%, (range, 0.0038–0.0095 wt.%) in the aortic plaques. However, it was not possible to analyze the absolute concentration of iron in the carotid plaques due to the concentration being below the measurement limit. The zinc concentration was variable between samples.

## Discussion

The process of vascular calcification shares many similarities with that of skeletal mineralization. However, while skeletal mineralization is a regulated process, induced by complex, well-timed developmental cues, vascular calcification is a pathological process that occurs in response to factors such as smoking, diabetes or uremia (1). The mechanisms underlying vascular calcification have yet to be elucidated. Vascular smooth muscle cells (VSMCs) are currently considered to be responsible for the formation of vascular calcification. Trion and van der Laarse (6), as well as Shroff and Shanahan (11), suggested that the apoptosis of VSMCs appeared to be a key factor in this process, while other factors, including cell-to-cell interactions between macrophages and VSMCs, lipids and plasma inorganic phosphate levels, modulated the calcification process. Proudfoot *et al*(12) suggested that the inhibition of apoptosis also inhibited vascular calcification, and that the stimulation of apoptosis promoted this process, providing a strong correlation between apoptosis and the initiation of vascular calcification. Ewence *et al*(13) suggested that calcium phosphate crystals initiated inflammation and led to VSMC apoptosis.

There have been numerous studies investigating the correlation between clinical events and vascular calcification (6,14,15). Certain studies suggested that patients with extensive calcification of the carotid plaques were less likely to suffer from stroke and transient ischemic attack (6,14). However, Nandalur *et al*(15) quantitatively evaluated the calcified atherosclerotic burden in cervical carotid arteries using multi-detector CT to analyze the correlation between the calcium scores and certain symptoms. It was observed that the carotid calcium scores were significantly correlated with symptom occurrence. Prabhakaran *et al*(16) also demonstrated that the presence of calcification in the carotid artery, as assessed using high-resolution B-mode ultrasound, was an independent predictor of vascular events, such as ischemic stroke, myocardial infarction and vascular death. According to epidemiological studies, the rupture of AAAs is correlated with the size of the aneurysm (17). It is generally acknowledged that AAA rupture occurs when the stress acting on the wall during the cardiac cycle exceeds the strength of the wall. Speelman *et al*(18) suggested that there was a weak correlation between aortic calcification and increased peak stress on the wall. However, Li *et al*(19) suggested that aortic calcification increased AAA peak wall stress and decreased the biomechanical stability of the AAA (19).

This study evaluated the concentrations of calcium and phosphate in the carotid artery and AAA specimens. To the best of our knowledge, there have been no previous studies investigating the concentration of mineral components in vascular calcification. Clinical studies have shown elevated circulating calcium, phosphate and calcium phosphate product levels to be correlated with increased vascular calcification in patients with end-stage renal disease (ESRD) (20). Furthermore, it has been demonstrated that elevated extracellular inorganic phosphate-induced VSMC calcification and osteogenic differentiation, with phosphate uptake into VSMCs, were mediated by the sodium-dependent phosphate co-transporter, pit-1 (21). The association between the concentrations of circulating calcium and phosphate ions and the concentrations of the components from specimens of vascular calcification require evaluation.

The levels of zinc and iron measured in this study were variable. Sullivan (22) first suggested in 1981 that the higher incidence of heart disease in men and post-menopausal women was associated with higher levels of stored iron. Although the impact of iron stores and iron therapy on cardiovascular risk is not well defined, ferritin is a marker of morbidity and mortality in patients undergoing hemodialysis, and the administration of high levels of intravenous iron increases the risks of hospitalization and death (23). Iron may contribute to cardiovascular complications through the effects on low-density lipoprotein (LDL) oxidation and endothelial dysfunction (24). In a series of patients from the Bruneck study, serum ferritin and LDL cholesterol exhibited a synergistic correlation with the progression of carotid atherosclerosis, suggesting that iron acted by promoting lipid peroxidation (25). The importance of peroxidation was further demonstrated by Salonen *et al*(26). Endothelial dysfunction, including the impairment of the action of nitric oxide, is a component of cardiovascular disease. In a study of patients with coronary artery disease and control subjects, iron chelation with a 1-h infusion of 500 mg desferrioxamine improved the blood flow response in the forearm to an endothelium-dependent vasodilating agent, metacholine (27). Leone *et al* reported that low serum Zn levels have been associated with increased cardiovascular mortality (28).

In conclusion, although the mineral components were analyzed in relatively few samples of aortic and carotid plaques in the present study, the concentration of calcium and phosphate in the aortic plaques was lower than that in the carotid plaques. The mineral concentrations of the plaques in the present study may be used as reference values for further studies on vascular calcification. An enhanced understanding of the complex mechanism regulating vascular calcification and the clinical correlation with vascular calcification may have therapeutic potential in the reduction of cardiovascular disease-associated morbidity and mortality. Further studies are required to elucidate the correlation between the mineral components and vascular calcification.

## Figures and Tables

**Table I:** Concentration of calcium (wt.%).

	Sample	
	
No.	Aortic plaques	Carotid plaques
1	19.3	4.43
2	2.10	16.1
3	9.71	11.6
4	7.09	12.1
5	10.2	9.20
6	16.6	18.2
7	14.1	
8	4.98	
9	4.35	
Mean	9.83	11.94

**Table II:** Concentration of phosphate (wt.%).

	Sample	
	
No.	Aortic plaques	Carotid plaques
1	8.53	5.25
2	0.97	7.83
3	4.3	5.17
4	3.08	5.6
5	4.46	4.28
6	7.42	8.33
7	6.03	
8	2.18	
9	1.86	
Mean	4.31	6.08

**Table III:** Concentration of iron (wt.%).

	Sample	
	
No.	Aortic plaques	Carotid plaques
1	0.0085	<0.01
2	0.0095	<0.01
3	0.0044	<0.01
4	0.0038	<0.01
5	0.0049	<0.01
6	0.0045	<0.05
7	0.0087	
8	0.0089	
9	0.0071	
Mean	0.0067	-

**Table IV:** Concentration of zinc (wt.%).

	Sample	
	
No.	Aortic plaques	Carotid plaques
1	0.018	0.008
2	0.0027	0.0081
3	0.0094	0.012
4	0.0058	0.0092
5	0.0082	0.004
6	0.0097	0.043
7	0.0085	
8	0.0066	
9	0.0059	
Mean	0.0083	0.014
